# Health Care Experiences of Patients Discontinuing or Reversing Prior Gender-Affirming Treatments

**DOI:** 10.1001/jamanetworkopen.2022.24717

**Published:** 2022-07-25

**Authors:** Kinnon R. MacKinnon, Hannah Kia, Travis Salway, Florence Ashley, Ashley Lacombe-Duncan, Alex Abramovich, Gabriel Enxuga, Lori E. Ross

**Affiliations:** 1 School of Social Work, York University, Toronto, Ontario, Canada; 2 School of Social Work, The University of British Columbia, Vancouver, British Columbia, Canada; 3 Faculty of Health Sciences, Simon Fraser University, Burnaby, British Columbia, Canada; 4 BC Centre for Disease Control, Vancouver, British Columbia, Canada; 5 Centre for Gender and Sexual Health Equity, Vancouver, British Columbia, Canada; 6 Faculty of Law & Joint Centre for Bioethics, University of Toronto, Toronto, Ontario, Canada; 7 School of Social Work, University of Michigan, Ann Arbor, Michigan; 8 Institute for Mental Health Policy Research, Centre for Addiction and Mental Health, Toronto, Ontario, Canada; 9 Dalla Lana School of Public Health, University of Toronto, Toronto, Ontario, Canada; 10 Department of Psychiatry, University of Toronto, Toronto, Ontario, Canada; 11 School of Social Work, McGill University, Montreal, Québec, Canada

## Abstract

**Importance:**

Medical education, research, and clinical guidelines are available to support the initiation of gender-affirming care for transgender and nonbinary people. By contrast, little is known about the clinical experiences of those who discontinue or seek to reverse gender-affirming medical or surgical interventions due to a change in gender identity, often referred to as *detransition*.

**Objective:**

To examine the physical and mental health experiences of people who initiated medical or surgical detransition to inform clinical practice.

**Design, Setting, and Participants:**

Using constructivist grounded theory as a qualitative approach, data were collected in the form of in-depth interviews. Data were analyzed using an inductive 2-stage coding process to categorize and interpret detransition-related health care experiences to inform clinical practice. Between October 2021 and January 2022, individuals living in Canada who were aged 18 years and older with experience of stopping, shifting, or reversing a gender transition were invited to partake in semistructured virtual interviews. Study advertisements were circulated over social media, to clinicians, and within participants’ social networks. A purposive sample of 28 participants who discontinued, shifted, or reversed a gender transition were interviewed.

**Main Outcomes and Measures:**

In-depth, narrative descriptions of the physical and mental health experiences of people who discontinued or sought to reverse prior gender-affirming medical and/or surgical interventions.

**Results:**

Among the 28 participants, 18 (64%) were assigned female at birth and 10 (36%) were assigned male at birth; 2 (7%) identified as Jewish and White, 5 (18%) identified as having mixed race and ethnicity (which included Arab, Black, Indigenous, Latinx, and South Asian), and 21 (75%) identified as White. Participants initially sought gender-affirmation at a wide range of ages (15 [56%] were between ages 18 and 24 years). Detransition occurred for various reasons, such as an evolving understanding of gender identity or health concerns. Participants reported divergent perspectives about their past gender-affirming medical or surgical treatments. Some participants felt regrets, but a majority were pleased with the results of gender-affirming medical or surgical treatments. Medical detransition was often experienced as physically and psychologically challenging, yet health care avoidance was common. Participants described experiencing stigma and interacting with clinicians who were unprepared to meet their detransition-related medical needs.

**Conclusions and Relevance:**

This study’s results suggest that further research and clinical guidance is required to address the unmet needs of this population who discontinue or seek to reverse prior gender-affirming interventions.

## Key Points

**Question:**

What are the health care experiences of adults undergoing medical and/or surgical detransition?

**Findings:**

In this qualitative study, 28 adults with heterogenous gender identities were interviewed about their experiences of detransition, including their health care encounters when discontinuing or reversing gender-affirming medical and/or surgical care. A majority of respondents reported no decisional regrets regarding prior gender-affirming interventions; however, participants frequently discussed health care avoidance, clinician stigma, and experiencing clinicians who lacked detransition-related clinical knowledge.

**Meaning:**

These findings suggest that clinicians may be insufficiently knowledgeable to meet the needs of this population, and further research and clinical guidance are required to better support people who detransition after pursuing gender-affirming health care.

## Teaser

This qualitative study examines the physical and mental health experiences of people who sought to discontinue or reverse gender-affirming medical or surgical interventions.

## Introduction

Transgender and nonbinary people experience to varying degrees gender dysphoria—distress related to an incongruence between one’s assigned sex at birth and their experience of gender.^1^ To alleviate gender dysphoria, some individuals pursue medical transition, which may include gender-affirming hormonal therapies and surgical procedures. For adults assigned male at birth (AMAB), medical or surgical interventions could include antiandrogens and estrogen therapy, facial feminization surgery, breast augmentation, orchiectomy, and vaginoplasty. For adults assigned female at birth (AFAB), available treatments include testosterone therapy, double mastectomy with male chest contouring, oophorectomy/hysterectomy, and a range of genital surgery options.^2^ In the past 10 years, adolescent and adult referrals to gender identity clinics in Canada, Europe, the US, and the United Kingdom (UK) have risen substantially,^3,4,5^ indicating that the number of individuals who medically transition is increasing.

Medical education, research, and clinical guidelines are available to support the initiation of gender-affirming care.^2,6,7,8,9,10,11,12,13^ By contrast, there is little clinical guidance when patients stop, or seek to reverse, gender-affirming medical interventions, referred to as detransition.^14^ Medical detransition involves ceasing or switching gender-affirming hormone therapy and/or surgical reconstruction or reversal.^15^ After medical detransition, individuals may continue identifying as transgender or nonbinary, or they may reidentify with their birth sex (eg, female or male).^16,17,18^ Clinicians who work in gender-affirming health care report fears of detransition as a patient outcome because of limited experience and concerns of harm and liability.^19^

Detransition appears to be an infrequent and occasionally temporary outcome, with estimates ranging from less than 1% to 13%, much of this variability owing to differences in methods, sampling, and conceptual definitions.^14,17,20,21,22,23^ At the high end of this range, Turban et al^18^ found that 13% of 17 151 transgender and nonbinary survey respondents reported a history of temporarily returning to their sex assigned at birth. Hall et al’s^22^ retrospective case-note analysis shows that out of 175 patients discharged from a gender identity clinic in the UK, 5 (2.8%) stopped transition; 6 (3.4%) paused their transition goals for social or health issues, and 12 (6.9%) reverted back to their assigned sex at birth. Of these 12, 4 patients were rereferred to the clinic, suggesting interest in resuming medical transition following detransition (eg, retransition). Individuals report detransitioning for reasons such as mental and/or physical health concerns,^15,17,21,22^ fertility preservation,^17^ change in political beliefs,^15^ shifting gender identity^14,15,16,17,21,24^ or understanding of one’s sexuality,^15,21^ social challenges living as a transgender person,^17,21^ pressure from a romantic partner or family member,^17^ employment discrimination,^17^ postoperative surgical pain,^25^ and career-related or financial concerns,^17,25^ among others. Some report regretting their initial gender transition, whereas others experienced it as an opportunity to explore and clarify their gender identity.^16,24,26^

There are scant studies that center patient experiences of detransition. One international survey of 237 self-identified detransitioners found 49% wanted information on stopping or changing hormones, and 15% required information on surgical reversals.^14^ However, 31% of the sample had not medically transitioned. Authors have called for qualitative research into detransition to understand this population’s everyday lives and their clinical needs.^16,22,27^ In response, we explored the physical and mental health experiences, gender identities, and other characteristics of a sample of detransitioners and retransitioners living in Canada. To our knowledge, this is the first qualitative study of detransition and retransition in the adult population.

## Methods

We selected constructivist grounded theory (CGT) methodology for its analytic utility in constructing new concepts grounded in research participants’ descriptive statements about their life events.^28^ The study protocol was reviewed and approved by the York University Research Ethics Board. Prospective participants were emailed a copy of the informed consent form to review in advance of the scheduled interview, and prior to initiating the interview participants provided verbal consent.

### Quality and Reflexivity Statement

We followed the Consolidated Criteria for Reporting Qualitative Research (COREQ) reporting guideline. CGT stresses that credibility is enhanced when researchers engage in reflexivity, acknowledging how their experiences can shape the research and interpretation process. Our team is composed of cisgender, trans, and nonbinary researchers. The interview guide was developed by the research team and each question was reviewed for concept accuracy by 2 project consultants with detransition/retransition experiences (eAppendix 1 in the Supplement). To elicit high quality data, all interviews were conducted by transgender or nonbinary interviewers with experience of accessing gender-affirming medical treatments, one of whom has medically detransitioned. Interviewers brought clinical social work training and/or qualitative interviewing skills, using rapport-building strategies with interviewees. These strategies included a preinterview eligibility screener conversation to confirm eligibility and answer all questions about the study posed by prospective participants. Within the interview itself, interviewers applied verbal agreements (eg, “Yes that makes sense.”), as well as clarifying participant responses by summarizing (eg, “To be clear, what you are telling me is…”). Following the recruitment and data analysis phases, participants were communicated an update regarding how their interview data were being mobilized, such as upcoming presentations and the sharing of anonymized quotes on social media. Participants were invited to opt out of having their personal quotes shared on social media. During data analysis and interpretation, we consulted with 2 additional detransitioners—both living in the US—and 2 Canadian psychiatrists working clinically with this population.

### Participant Selection and Interview Procedures

Participants were sampled using purposive and snowball sampling. To be eligible, participants had to be aged 18 years and older, living in Canada, able to participate in an interview conducted in 1 of the 2 official national languages—English or French—and self-identify as detransitioning, retransitioning, detrans, retrans, reidentifying, experiencing a shift in gender identity after initiating transition, or having stopped transition. Transition included social, legal, or medical gender transition. A broad definition of detransition consistent with existing literature was used to maximize heterogeneity in the sample. We used language neutral in tone to invite a diverse range of individuals (see eAppendix 2 and 3 in the Supplement for study advertisements). Recruitment flyers were promoted using Twitter, Facebook, Instagram, TikTok, and private internet-based detransition support groups. Flyers were sent to clinicians known to serve trans people and those pursuing detransition in 6 urban centers across Canada. Following purposive and snowball sampling, participants who discussed gender transition reversal and/or reidentifying with their birth sex—in particular—were encouraged to share the research flyer among their networks.

Data were collected between October 2021 and January 2022; 42 individuals expressed interest in the study and were invited for eligibility screening via telephone or Zoom. Of those who initially contacted the study, 14 did not partake because they: (1) were unresponsive to follow-up emails; (2) did not live in Canada; or (3) had never discontinued or reversed a gender transition and were therefore ineligible. Semistructured, in-depth one-on-one virtual interviews (n = 28) were conducted with participants who met eligibility criteria and all participants completed the full interview and demographic questions. Two interviews were conducted in French. The Zoom platform enabled geographic sampling of participants living in urban, suburban, and rural regions of Canada. Zoom interviews are a useful alternative to in-person interviews in qualitative research and offer a level of perceived anonymity that can be beneficial for data collection on sensitive topics.^29,30^ Interviews were between 50 to 90 minutes, audio-recorded, and transcribed verbatim. Participants received a $30 CAD (US $23) electronic gift card. To understand the demographic characteristics and heterogeneity of the sample, participants were asked how they self-described their race and/or ethnicity, assigned sex at birth, and sexual orientation. These categories were constructed by directly following participants’ own language. See the eTable in the Supplement for sample characteristics.

### Data Analysis

In-depth narrative interview data were analyzed inductively and involved 2 phases of coding. Following 20 interviews, K.M. and G.E. pursued initial coding in which data were classified into preliminary themes and a coding framework. We leveraged this initial process to assess saturation, a measure of analytical rigor in qualitative research which confirm no new themes emerge from the data. In the second stage of coding, the final 8 interviews served to confirm theoretical saturation across the data set, and to finalize the coding framework (eAppendix 4 in the Supplement). All transcripts were read and reread by a minimum of 2 authors (K.M., G.E., L.R.) and coded in Dedoose.

Because each participant experienced a shift in how they understood or expressed their gender after initiating gender transition, alongside the illustrative quotes we present participants’ assigned sex at birth together with a description of their current gender. See the eFigure in the Supplement for current gender identities.

## Results

Among the 28 participants, 18 (64%) were assigned female at birth and 10 (36%) were assigned male at birth. Twenty participants (71%) were between the ages of 20 to 29 years, 6 (22%) were between 30 to 39 years, and 2 (7%) were aged 40 years or older. When asked demographic questions about race and ethnicity, participants were provided the option to use preferred language; 2 (7%) identified as Jewish and White, 5 (18%) identified as having mixed race and ethnicity (which included Arab, Black, Indigenous, Latinx, and South Asian), and 21 (75%) identified as White. One participant’s transition and detransition was exclusively social and was therefore removed from the analysis of medical detransition. Medical transition was initiated by 27 participants at a wide range of ages (15 [56%] were between the ages of 18 and 24 years and 6 (22%) were between ages 25 and 29 years).

Participants discussed multiple reasons for discontinuing or reversing their gender transition and these intersected with experiences of detransition-related care and health care encounters. Factors included physical and/or mental health concerns, surgical complications and/or postoperative pain, unsupportive parents or romantic partners, employment discrimination, difficulty accessing clinical appointments or gender-affirming surgery due to COVID-19 lockdowns or because they lived in low-resourced regions of Canada, among other reasons consistent with existing literature. Participants experienced the medical detransition process as physically and psychologically challenging and health care was perceived as suboptimal because of detransition-related stigma, or clinicians lacked information. Illustrative quotes supporting these findings are shared here and in the Table.

**Table.** Demonstrative Quotes

Theme	Illustrative quotations
Gender-affirming hormonal therapy cessation or switching and clinical care needs	“I’m sort of in the detrans Twitter circle… I was really helped by other detransitioners, because there wasn’t [detransition-related medical] information…” (Participant #10, AFAB, female)
	“Once I transitioned in the system from youth to adult care, at 18, my family doctor was just prescribing my testosterone… So, I stopped taking it, didn’t tell him. I stopped taking it cold turkey… I started going to a youth group for queer youth, and that’s where I met a bunch of other trans people my age, including trans-masculine people who did not present in extremely binary ways, who did not speak about gender the way I was taught to speak about gender, did not dislike themselves as much as I did, and I am still friends with most of those people… And I think now, what was going on in my head was that I didn’t think that I could be part of something—something being the trans community—I didn’t think I really could be part of something that would require me to love myself that much. So I detransitioned… I returned to presenting as a butch lesbian.” (Participant #13, AFAB, nonbinary trans)
	“[T]he woman I was talking to—the one with the series of YouTube videos—I met her a few days after the last dose of testosterone. She was like ‘Oh, well have you talked to a doctor about this?’ I was like, ‘No, I have no idea what’s going to happen. I’m just not going to do it anymore.’ And she was like, ‘OK, so you’re going to want to look out for, as it leaves your system.... you’re going to have a week or two where you’re super tired all the time. It’s going to be really intense. You’re going to be exhausted.’ …I didn’t know what to expect, except for what the other detransitioned women on the internet had told me.” (Participant #05, AFAB, trans)
	“[When I began my transition, my] doc sent me to Dr [name of doctor], and that was inconvenient for me. I mean, it’s a 2 ½ to 3-hour drive there, and the parking and everything, taking time off of work. And so, yeah, it was just, it was a nightmare in a sense. But I was really keen and eager, and I was so happy to have my prescription and begin hormones finally. And I went from euphoria, yay me, to like, it’s the end of the world kind of feeling now [detransitioned]… everything went into [COVID-19] lockdown… But at this point I just thought it was safer to, you know, not mess around with my dosages myself… and reanalyze once this all COVID thing clears up and goes away, or whatever. And hopefully start again, yeah.” (Participant #02, AMAB, gender-fluid nonbinary)
	“I think I still felt like a little bit of guilt and shame about the whole [detransition]. I don’t really entirely know. I think it’s just always revolved around not wanting other [trans] people to look bad.” (Participant #20, AFAB, female)
	Participant: “I’m really lucky… (Doctor’s name) and she’s been my family doctor, since I started my transition basically. So, she’s very knowledgeable. She works with lots of trans folk. And yeah, so I’m really lucky in that in that regard.” Interviewer: “And so, how did you feel at the time when you started taking estrogen [during detransition-related hormonal switching]?” Participant: “Definitely some weird changes over time. The mental part of it was actually the hardest. So, there’s definitely, dealing with a wider breadth of emotional experiences. Being on testosterone for so long, I think... You get kind of, like flat, you know… Because I don’t have ovaries. So, there’s no other hormones in my body.” (Participant #09, AFAB, undefined gender identity)
	“I wish [detransition] was something I could have done on my own, but I had to go see a doctor because I would have had to switch from testosterone to estrogen. I did go see a doctor and I had a pretty bad experience… So, I ended up having to see another one… The first doctor I went to... She was not very tactful, I guess you could say... We only have 2 gender specialist doctors here in my city. We don’t have a lot. She wasn’t very tactful. She made comments about how I should have thought about [my initial transition] harder.” (Participant #14, AFAB, female)
	“The first doctor I went to—and the second doctor—both didn’t have a clue what was going on… What’s going to happen when you go off testosterone and back on estrogen? …I was struggling with a lot of dizziness and sick feelings… So, I feel like more information [is needed] around specifically people who need to get off testosterone to go back onto another [hormone]. Because I’ve had [oophorectomy and] hysterectomy.” (Participant #14, AFAB, female)
	“I’ve been going to the same doctor since I started hormonal care…They were very supportive of my gender transition. They wrote letters for gender confirmation surgery [orchiectomy]. When I went in, I don’t think I framed [detransition] clearly because I was a bit nervous how they would react. I was like ‘I think I need to go on testosterone for these medical issues.’ And they were like, ‘This is outside of my practice. You should see an endocrinologist.’ So, when I saw the endocrinologist he was very helpful and I was much more clear. I was like ‘I want to start taking testosterone anyway. I want to change the gender trajectory I’m on.’ …He’s like “OK we’ll do a blood test. Come back in a few weeks. We’ll talk about doing testosterone.” …Neither he nor the other endocrinologist at that clinic had treated a transgender patient who needed to go on testosterone to detransition… But I’ve gotten good support and the same doctor’s been helping me taper off the feminine hormone and get ready to switch to the testosterone.” (Participant #17, AMAB, gender-fluid male)
	“I’m like, ‘Is it the estrogen that’s throwing me off and I’m all over the place and, you know, my brain is just, you know, going crazy?’ So nobody was giving me any answers so I said ‘Oh, well that’s it. I can’t do this anymore. I can’t live like this for the rest of my life.’ So I went off estrogen and then things started improving. When I went to the endocrinologist at that point, I said, ‘Hey can I just be nothing? Like why do I need testosterone or estrogen?’ And sometimes I think I’d prefer that feeling of that in-between. I still feel like that now that I’m back on testosterone for the last couple years, I have this low-level anxiety that I’m just tense all the time on testosterone. I think that’s what drove me to transition in the first place, right. Like I have that back now, from prior to [year] when I transitioned.” (Participant #28, AMAB, nonbinary male)
	“That is what my transition looked like. It was a hot mess. I was a hot mess. But I know people that it has benefited. I know people that [transition] has helped, with their mental health and they’ve done better. But they started to do better earlier in their transition. So, like as they were on hormones and they started to pass, their mental health was improving... And that pattern wasn’t seen in me. The farther I got into transition, the worse my [borderline personality disorder] symptoms and my presentation was.” (Participant #10, AFAB, female)
Current perspectives on past gender-affirming hormones and surgeries	Interviewer: “Do you feel regretful of having had the double mastectomy?” Participant: “Some days I do, some days I don’t. Some days it doesn’t bother me, other days it bothers me. It just depends.” Interviewer: “Are you thinking of getting breast augmentation surgery in the future?” Participant: “No. No. I’m just going to leave myself alone. It’s part of my story. It’s part of my journey. That’s the way I try to look at it.” (Participant #22, AFAB, female)
	“My body’s changed, right. I have small breasts that estrogen created, and I still have a vagina and I have no plans to change that. I may have the breasts reduced because there is a bit of self-consciousness there with people seeing me as male… I’m not a hundred percent sure on that. I don’t want to put myself through any more surgery. As far as my body goes, I’m OK with it.” (Participant #28, AMAB, nonbinary male)
	“As I’ve said, I’ve got my beard still... the facial hair doesn’t go away once it starts growing. And I wouldn’t have that if I had just stayed in my estrogen-based body the whole time. I had some physical changes from testosterone. Like my voice did drop, I had like genital changes. And I’m very happy with all of that stuff. The reason that I’m comfortable not being on testosterone is because I had those changes and those are things that aren’t going to reverse themselves. So, I have a really gender-ambiguous body. And that’s not something that I would have been able to have if I hadn’t taken the steps that I did.” (Participant #16, AFAB, nonbinary)
	Participant: “Well, I identify as a cisgender woman. But like, I feel like my experience having detransitioned is different than a lot of cis women’s. Like in public, I don’t pass as a cis woman. So that’s kind of like.... It’s kind of a unique experience to, like, be a cis woman who doesn’t pass [as a cis woman]. And so, I’m usually read as a trans person even though I don’t identify as a trans person anymore…” Interviewer: “Yeah. And so now looking back, now that you have detransitioned, do you feel like starting hormones was the right or the wrong decision at the time?” Participant: “I actually I feel like it was really the right decision. Everyone assumed that I would regret it because I don’t pass for a cis woman anymore. But, at the time, that was absolutely what I knew I had to do. I’m actually not upset about any of the permanent changes it had on my body.” (Participant #07, AFAB, cis woman)
	“I think it [transition] was definitely the right decision… The only thing I wish that I would have known is what going off of hormones looked like. Because it took about a year for the full changes, and my voice got higher again, which I did not expect. And I lost most of my body hair. I was extremely hairy. But I can still grow a full beard, so it’s strange and I definitely don’t regret it, but I wish I would have had more information [on stopping hormones].” (Participant #03, AFAB. nonbinary trans)
	“Like after I got over a lot of these initial feelings of like, ‘I don’t know what to do, I made a mistake, I didn’t make the right choice, where do I go from here? What if my next decision is the wrong one?’ I started to feel none of the choices that I’ve ever made in my transitions have ever been the wrong ones, because they were the right choices to make at the time that I made them. (Participant #01, AMAB, gender-fluid nonbinary)
	“There’s always someone waiting to pounce on [detransition] and say ‘Oh, see, trans people are fake, they’re just faking it,’ and it’s like, ‘No, if I had’ve had better access to trans health care I wouldn’t have made decisions that I would have regretted.’ Because I’ve basically had to prove that I was ‘trans enough’ [by taking testosterone] in order to access [surgery] that I needed in order to feel OK. And like I don’t regret top surgery [chest masculinization] for a minute. Like it’s just the testosterone and how that presentation worked.” (Participant #12, AFAB, gender-fluid)
	“I’ve had at various points feelings of regret about transition, both in terms of regretting going on hormones or regretting how soon I decided to go on hormones… I’m like, ‘Maybe I want to undo that, maybe I want to just fully detransition and just be a guy again or change my name to a less feminine name or something’… And it happens most often when I’m having bad social encounters, like if I meet a bunch of people and constantly get gendered as male, it hits me real bad that day. Or if I’m thinking about future jobs or future careers that I’m trying to get into and I’m thinking how hard is this going to be to navigate if I am trans or if I am nonbinary, that’s when I start thinking maybe I’ll just detransition, maybe that’ll be easier.” (Participant #25, AMAB, nonbinary)

Abbreviations: AFAB, assigned female at birth; AMAB, assigned male at birth.

### Gender-Affirming Hormonal Therapy Cessation or Switching and Clinical Care Needs

Discontinuing gender-affirming hormone therapy was characterized by participants as a medical detransition event and disengagement from health care was frequently reported. Many stopped hormone treatments “cold turkey” without psychological supports or medical supervision. Participants did not have a clear understanding of what health implications to expect when stopping gender-affirming hormones. Rather than rely on clinicians who were often experienced as a source of distrust, they turned instead to online detransition networks and social media.

“I actually just stopped talking to them [clinicians]… I felt like they were going to be mad at me [for detransitioning]… I didn’t refill my next prescription. I didn’t get the bloodwork tests. I just stopped seeing my endocrinologist when I decided to detransition... So unfortunately that involved quitting testosterone cold turkey… I was in a bit of a low [mood] just kind of weaning off and getting my hormones back to normal. I kept looking after myself and it passed. I, around that time, too, stopped seeing my therapist. I had like almost no supports when that was happening [detransition]. So I just really kind of looked online like see what kind of groups there were for people who had detransitioned.” (Participant #20, AFAB, female)

During her initial medical transition, participant #20 noted having overall positive relationships with her health care clinicians and therapist. Yet she felt “guilt and shame” about detransitioning and was concerned that her initial transition would be misinterpreted by clinicians as a “mistake” or through a lens of “regret” which was inauthentic to her feelings.

Overall, the medical detransition process was experienced as physically and psychologically challenging and participants described clinicians who lacked sufficient information. Even those reporting good access to clinical supports noted challenges with hormone therapy cessation, such as resumed gender dysphoria. For some, gender-affirming hormonal therapy had substantially reduced symptoms of gender dysphoria but they discontinued this treatment for other health considerations.

“I have a family history of breast cancer. Both my grandmother and mother and also on my father’s side… [To reduce risk for breast cancer] Went off [the anti-androgen] and then tapered off the estrogen. But it was over a period of less than a month. And I saw a lot of changes in my body that were difficult, [gave] me a lot of dysphoria. Facial hair in particular started growing back in full force. So that was the most difficult part. It was difficult giving up some of the good parts about estrogen. There was a bit of a mourning period there and there still is.” (Participant #15, AMAB, nonbinary)

Other participants disclosed that their parents or family circumstances explicitly forced, or implicitly encouraged, detransition—typically without medical supports. Each of these participants sought care again later.

“It was a couple weeks after I turned 18 that [the clinic] put me on the hormones. I was then able to be on hormones for a while without my parents’ knowledge. But since I had come out [as trans] before, [my parents] knew what to look out for and started going through my stuff [at home]. So one day [my parents] found my HRT [hormone replacement therapy] and made me stop taking it and quit contact with the doctor I was seeing in the clinic… I was [on hormones] for about four months and then I was caught and I stopped for a year.” (Participant #24, AMAB, transgender woman)

This participant returned to the same clinic 1 year later and noted that during this period of care disengagement there was no follow-up from the clinic. A lack of follow-up from clinicians after disconnecting from care was a consistent theme described by many participants.

Given the need to switch hormone therapy (rather than discontinue), most participants who had past gender-affirming gonadectomies reported medical supervision. The care they received was described as “bad” because clinicians were inadequately knowledgeable or judgmental. This amplified some participants’ internal emotional struggles with feeling shame, and it confirmed their fears of clinician judgment. Most participants described access to gender-affirming care as scarce in the first place, which in turn restricted their detransition-related care options.

### Current Perspectives on Past Gender-Affirming Hormones and Surgical Procedures

Participants discussed a range of divergent perspectives about their history of gender-affirming hormones and/or surgical procedures and how these intersected with shifts in their gender identities over time. Of the 27 participants who pursued gender-affirming hormones and/or surgical procedures, 18 (67%) expressed no regrets and/or positive feelings associated with past gender-affirming interventions. Six (22%) experienced regret and felt that medical transition was the wrong pathway for them. Three (11%) expressed ambivalent feelings such that they do not regret their decision to medically transition but would have slowed the process, or feeling dissatisfied with their bodies some days and content other days. Of those who were regretful or ambivalent, many discussed a need for psychological and social supports.

One participant sought surgical reversal to address both feelings of regret and surgical complications and pain sustained over several years. “I had complications with [genital] surgery which were bad… I was dealing with a complication that was painful for 6 years, 7 years… I went back to the clinic. And this same doctor who had done the original surgery.... They fixed the complication. Then I had another complication that I lived with for a long time. And just this [past] summer I went and had that fixed. And while I was there, I told the surgeon that I was detransitioning and had been detransitioned for about a year and I asked if he could... Well, basically undo some of the surgery. And he did… He did more than he had to. That was a good interaction for me… And he didn’t ask many questions about it. I don’t know why. But anyway, it went really well and I’m pleased with how it how it turned out.” (Participant #9, AFAB, undefined gender identity)

Others felt neutral or satisfied with their bodies and physical changes caused by gender-affirming medical interventions. Some noted preference for their current physical bodies which had been hormonally or surgically altered, including individuals who no longer identified as transgender or nonbinary and who had returned to living socially in their assigned sex at birth.

“The few changes I did have, when I was on hormones and stuff, I do not really regret… And I do actually like the changes [from hormones]… I wasn’t too fond of how my voice sounded when growing up. So hormones did help with that.” (Participant #20, AFAB, female)

## Discussion

At the population level, hormonal and surgical interventions are effective in improving transgender people’s gender dysphoria, mental health outcomes, and social well-being.^3,11,31,32^ Yet, at the individual level it is not always possible for patients—or their clinicians—to predict with absolute certainty how treatments will be experienced.^19^ Our study highlights clinical practice gaps when medical detransition occurs.

Our analysis points to a concerning trend of disengagement from psychosocial and medical supports when initiating hormonal cessation—a period marked by increased need for care owing to participants’ reports of changes in energy and mood, worsened gender dysphoria, and overall uncertainty about detransition. We note that robust clinical outcomes research is needed to understand further the physiological and psychological effects of discontinuing or switching gender-affirming hormones, as data to triangulate these participants’ accounts are presently unavailable. Still, our data offer qualitative interpretation to Hall et al’s^22^ retrospective case-note analysis, which raised questions about gender identity clinic disengagement and detransition or retransition. Our data clarify disengagement may occur for several complex reasons, such as having poor access in the first place, or feeling shame and anticipating stigma. Participants avoided clinicians and detransition-related mental health care despite reporting psychological distress. Health care avoidance behaviors associated with clinician judgment and stigma are broadly reported by lesbian, gay, bisexual, and transgender populations.^33,34,35,36^ Clinicians providing gender-affirming care must be careful to avoid shaming patients who are pursuing hormonal cessation or switching or surgical reversals and instead strive to address current mental and physical health needs. Failure to do so will likely only exacerbate such disengagement behaviors. We recommend that when discussing treatment options for gender dysphoria, clinicians state explicitly that if the patient chooses to discontinue any gender-affirming interventions they will continue to support the patient.

Our study also included participants who experienced family pressure to detransition, which in turn contributed to medically unsupervised hormonal cessation and later re-initiation—a finding consistent with Turban et al’s^17^ study indicating some transgender and nonbinary people report a history of externally-influenced detransition. In our sample this occurred among younger transgender people who were living with unsupportive families. Clinicians ought to be attuned to patients’ family environments and disconnection from care.

Our findings confirm previous research indicating a clinical need for information on stopping or changing hormones.^14^ Yet, our analysis shows clinicians may be insufficiently prepared. Given the absence of clinical guidance on detransition/retransition, this is an unsurprising finding. Our data also uncover widely divergent perspectives on past gender-affirming medical interventions and how those relate to current gender identities, with a majority of participants expressing no decisional regret. As such, clinicians should investigate patients’ feelings and care needs without assuming people experience their initial transition as a “mistake”. Those with ambivalence or regrets may require formal psychosocial supports offered from an affirming and nonjudgemental approach. Relatedly, participants sourced peer-based medical advice and support from online detransition networks to address unmet clinical care. This is consistent with existing research showing transgender people seek and provide peer-based mental health support^37^ and engage in peer-to-peer health information sharing.^38^

### Limitations

Several weaknesses and strengths of our study warrant consideration. Study limitations include recall bias, as most participants described life events that spanned over ten years. Moreover, we did not interview clinicians to triangulate participants’ accounts of their health care experiences. One strength of our study is its heterogenous sample in terms of age range, past and current gender identities, and age of initiating transition. Another strength is its national scope. As health care delivery is affected by geopolitical context, studies^15,16^ that sample detransitioners internationally may be insensitive to local medical education curricula, the impact of regional health care funding on service delivery, and gender-affirming care legislation.

## Conclusions

We responded to calls for qualitative research on detransition,^22,27^ analyzing in-depth narratives spanning decades of life experiences and dynamic gender transitions. We found accounts of people ceasing or reversing gender-affirming treatments with sub-optimal care, warranting more robust clinical practice knowledge. Future research studies should examine clinicians’ knowledge of detransition and determine sources of stigma. This study’s results show that further research and clinical guidance is required to address the unmet needs of this population who discontinue or seek to reverse prior gender-affirming medical interventions.

## Notes

Supplement. **eFigure.** Current Reported Gender Identity **eTable.** Demographics **eAppendix 1.** Sample Interview Questions **eAppendix 2.** Study Advert (English) **eAppendix 3.** Study Advert (French) **eAppendix 4.** Coding Framework – Detransition/Retransition and Healthcare Needs
