# Supplementary material for: Health Care Experiences of Patients Discontinuing or Reversing Prior Gender-Affirming Treatments
Source: JAMA Netw Open. 2022 Jul 25;5(7):e2224717. doi: 10.1001/jamanetworkopen.2022.24717 (PMC9315415; doi:10.1001/jamanetworkopen.2022.24717)
Supplement: Supplement. — eFigure. Current Reported Gender Identity eTable. Demographics eAppendix 1. Sample Interview Questions eAppendix 2. Study Advert (English) eAppendix 3. Study Advert (French) eAppendix 4. Coding Framework – Detransition/Retransition and Healthcare Needs [file jamanetwopen-e2224717-s001.pdf]

Supplemental Online Content

MacKinnon KR, Kia H, Salway T, et al. Health care experiences of patients discontinuing or reversing prior gender-affirming treatments. *JAMA Netw Open.* 2022;5(7):e2224717. doi:10.1001/jamanetworkopen.2022.24717

eFigure. Current Reported Gender Identity

eTable. Demographics

eAppendix 1. Sample Interview Questions

eAppendix 2. Study Advert (English)

eAppendix 3. Study Advert (French)

eAppendix 4. Coding Framework – Detransition/Retransition and Healthcare Needs

This supplemental material has been provided by the authors to give readers additional information about their work.

© 2022 MacKinnon KR et al. *JAMA Network Open.*

eFigure. Current Reported Gender Identity

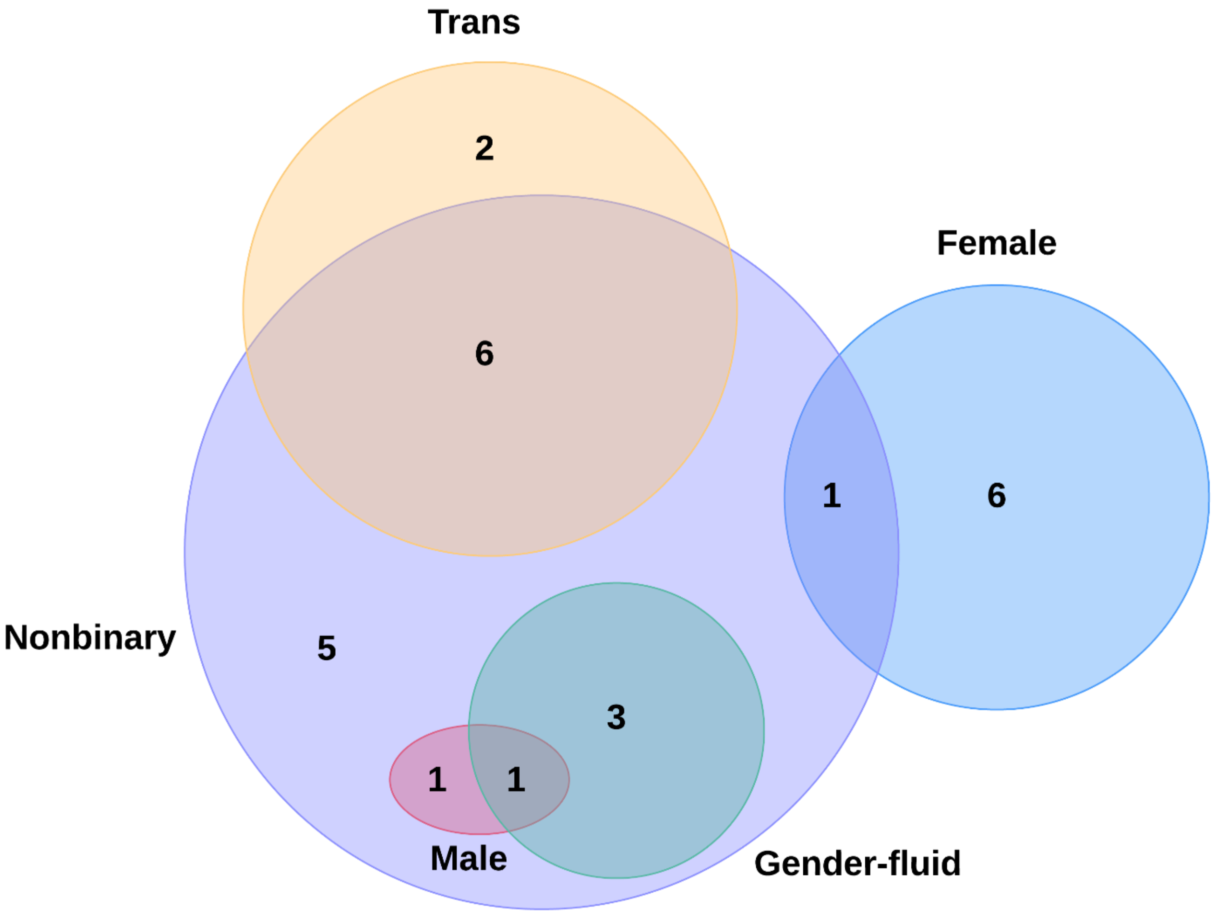

Notes: Participants were asked to describe, in their own words, their current gender identity. Most participants applied more than one gender identity label, and some indicated a preference for assigned sex at birth over gender (e.g., “female” rather than “woman”). Three (3) participants were undecided about current gender identity and are therefore not reflected in this Figure. The sum of the numbers appearing in a circle corresponds to the number of study participants who self-labelled with this descriptor. The largest circle holds a total of seventeen (17) participants who identified as “nonbinary”. Of these, six (6) also identified as “trans”. Some nonbinary participants further described themselves as “male” or “female”. Six (6) participants identified as “female”. This complex overlap in participants’ gender identities after detransition is similar to the sample of 100 medical detransitioners presented by Littman (2021).

© 2022 MacKinnon KR et al. *JAMA Network Open.*

eTable. Demographics

	No. (%)
Age (n=28)	
20-29	20 (71%)
30-39	6 (22%)
40+	2 (7%)
Age of Medical Transition (n=27)	
15-17	2 (7%)
18-24	15 (56%)
25-29	6 (22%)
30-39	3 (11%)
40+	1 (4%)
Social transition, without medical transition (n=1)	1 (100%)
Province	
Alberta	1 (3%)
British Columbia	4 (15%)
Manitoba	1 (3%)
New Brunswick	3 (11%)
Newfoundland	3 (11%)
Nova Scotia	2 (7%)
Ontario	7 (25%)
Quebec	7 (25%)
Urban/Suburban/Rural (n=28)	
Rural	4 (14%)
Suburban	1 (4%)
Urban	23 (82%)
Assigned Sex at Birth (n=28)	
Female (AFAB)	18 (64%)
Male (AMAB)	10 (36%)
Sexual Orientation (n=28)	
Asexual	1 (3%)
Bisexual/pansexual	9 (32%)
Gay/lesbian/homosexual	10 (36%)
Heterosexual	1 (4%)
Queer	7 (25%)
Race/Ethnicity (n=28)	
Jewish (White)	2 (7%)
Mixed (includes Black, Indigenous, Arab, Latinx, & South Asian)	5 (18%)
White	21 (75%)
Income Type (n=28)	
Employed	13 (47%)
State income support	10 (36%)
Student	3 (11%)
Unemployed	2 (7%)

© 2022 MacKinnon KR et al. *JAMA Network Open.*

eAppendix 1. Sample Interview Questions

1. To start, can you tell me about your current sex/gender identity and expression?
   Probe: How is this sex/gender identity/expression different from your previous one(s)?

2. Can you tell me more about your previous gender identity of ______?

3. To affirm a trans or nonbinary identity, what steps did you take to transition the first time?
   Probe: Did you see a healthcare professional like a doctor, social worker, or psychologist?
   Probe: What type of pathway did you go through to access gender-affirming healthcare?
   Probe: At the time, how did you feel about physically transitioning? (This could have included taking hormones, having surgeries, or other physical interventions such as hair removal).

4. Looking back, do you feel like transitioning was the right or wrong decision for you at the time?

5. What steps did you take to de/retransition, or to stop transitioning? (use participant's own language)
   Probe: At the time, how did you feel about physically detransitioning/retransitioning or reversing your transition?

6. Did you see a healthcare provider in connection with your de/retransition?
   Probe: How did your ___(provider)__ respond?
   Probe: Did you return to the same provider from the past?
   Probe: Did they seem knowledgeable about de/retransition?
   Probe: Did they seem supportive of your decision to de/retransition?

7. What is your main recommendation to care providers, like a doctor, psychologist, or a social worker, who want to provide better care and support people who are de/retransitioning? (use participant's own language).

© 2022 MacKinnon KR et al. *JAMA Network Open.*

eAppendix 2. Recruitment Flyer (English)

Did you experience a shift in your gender identity after you transitioned or did you stop transitioning? *
If so, we want to hear about your experiences and ideas about retransitioning and/or detransitioning

You may be eligible to volunteer for a 60-minute research interview if you:

- Are age 18 or older
- Live in Canada
- Can participate in a virtual interview conducted in English

AND

You self-identify as **one or more** of the following:

- Detrans/detransitioned/detransitioning
- Retrans/retransitioned/retransitioning
- Discontinuing transitioning
- Reidentifying with cisgender identity
- Experiencing a shift in gender identity after transitioning

Study participants will receive a $30.00 Canadian gift card to value their time and input.

The purpose of this study is to develop more understanding and supports for people who de or retransition.

Contact:
Kinnon R. MacKinnon, MSW, PhD
School of Social Work, York University
Phone: 416-736-2100 x 66333
E-mail: kinnonmk@yorku.ca

*This study defines “transition” to include a broad range of transitioning, such as social, legal, and medical transition. These may include: social name change or using new pronouns, legally changing your name or gender marker, or using medical treatments to express your gender.

This study is funded by the Social Sciences and Humanities Research Council and York University.

© 2022 MacKinnon KR et al. *JAMA Network Open.*

eAppendix 3. Dépliant sur le recrutement (français)

Avez-vous vécu un changement dans votre identité de genre après votre transition ou avez-vous arrêté votre transition ? *

Si oui, parlez-nous de vos expériences et de vos idées concernant la retransition et/ou la détransition.

Vous pourriez être éligible pour participer à un entretien de recherche de 60 minutes si vous :

- Êtes âgé de 18 ans ou plus
- Vous vivez au Canada
- Pouvez participer à une entrevue virtuelle en français

ET

Vous vous identifiez comme **une ou plusieurs** des personnes suivantes :

- Détrans / détransitionné.e
- Retrans / retransitionné.e
- Cesser une transition
- Réidentifier à l'identité cisgenre
- Vivre un changement d'identité de genre après une transition

Les participants à l'étude recevront une carte-cadeau canadienne de 30 $ pour leur temps et leur contribution.

L'objectif de cette étude est de mieux comprendre et de soutenir les personnes en dé/retransition.

Contact :
Kinnon R. MacKinnon, MSW, PhD
École de travail social, Université York
Téléphone : 416-736-2100 x 66333
Courriel : kinnonmk@yorku.ca

* Cette étude définit le terme "transition" comme incluant un large éventail de transitions, telles que la transition sociale, légale et médicale. Il peut s'agir d'un changement de nom social ou de l'utilisation de nouveaux pronoms, d'un changement légal de nom ou de genre, ou d'interventions médicales pour exprimer son genre.

Cette étude est financée par le Conseil de recherches en sciences humaines et l'Université York.

© 2022 MacKinnon KR et al. *JAMA Network Open.*

eAppendix 4. Coding Framework – Detransition/Retransition and Healthcare Needs

1. **Experiences of identifying as trans and affirming trans identity**
   a. Medical gender transition
   b. Legal gender transition
   c. Social gender transition
   d. Costs with gender transition

2. **Detransition experiences**
   a. Factors contributing to detransition
   b. Family and friends
   c. Encounters with clinic and care providers relating to detransition
   d. Medical supervision with medical detransition
   e. Retransitioning (re-identifying as trans/nonbinary and/or restarting HRT)
   f. Detransition and mental health
   g. Detransition and physical health

3. **Recommendations for healthcare providers and service delivery**
   a. Destigmatizing detransition and gender fluidity
   b. Understanding nonbinary identities and support gender exploration
   c. Language recommendations
   d. Mental health supports

© 2022 MacKinnon KR et al. *JAMA Network Open.*
